# C‐reactive protein improves the ability to detect cardiometabolic risk in mild‐to‐moderate obstructive sleep apnea

**DOI:** 10.14814/phy2.13454

**Published:** 2017-09-26

**Authors:** Jordan Gaines, Lan Kong, Menghan Li, Julio Fernandez‐Mendoza, Edward O. Bixler, Maria Basta, Alexandros N. Vgontzas

**Affiliations:** ^1^ Department of Psychiatry Sleep Research and Treatment Center Pennsylvania State University College of Medicine Hershey Pennsylvania; ^2^ Department of Public Health Sciences Pennsylvania State University College of Medicine Hershey Pennsylvania

**Keywords:** Biomarker, hyperglycemia, hypertension, inflammation, metabolic syndrome, obstructive sleep apnea

## Abstract

Obstructive sleep apnea (OSA), particularly in the mild‐to‐moderate range, affects up to 40% of the adult general population. While it is clear that treatment should be pursued in severe cases of OSA, when and how to best treat OSA in the mild‐to‐moderate range remains complicated, despite its high prevalence. The aim of this study was to compare the relative utility of apnea/hypopnea index (AHI) versus a biomarker of inflammation, C‐reactive protein (CRP), in identifying the presence and severity of hypertension and hyperglycemia. Middle‐aged (*n* = 60) adults with mild‐to‐moderate OSA (AHI between 5 and 29 events per hour) underwent 8‐h polysomnography, a physical examination including measures of blood pressure and body mass index, and a fasting morning blood draw for glucose and CRP. CRP levels were associated with greater odds for having hypertension and hyperglycemia compared to AHI. Receiver‐operating characteristics (ROC) curves revealed that adding CRP to standard clinical factors (age, sex, and BMI) yielded moderately good to strong risk models for the disorders (AUC = 0.721 and AUC = 0.813, respectively). These preliminary findings suggest that including a measure of CRP improves the ability for clinicians to detect cases of mild‐to‐moderate OSA with true cardiometabolic risk, with implications in improving prognosis and treatment within this clinically gray area.

## Introduction

Obstructive sleep apnea (OSA) is a prevalent sleep disorder characterized by intermittent hypoxia during sleep due to obstruction of the upper airway. OSA severity is quantified by the apnea/hypopnea index (AHI), defined as the number of apneas (complete obstruction of the upper airway for at least 10 sec indicated by no oronasal airflow, despite breathing effort) and hypopneas (partial airway obstruction, with either an associated 3% reduction in blood oxygen saturation or an arousal) summed per hour of sleep (Iber et al. 2007; American Academy of Sleep Medicine, 2014).

While it is clear that treatment should be pursued in more severe cases of OSA (AHI ≥ 30), when and how to best treat OSA in the mild (5 ≤ AHI < 15 events/h) to‐moderate (15 ≤ AHI < 30 events/h) range remains a clinically gray area, particularly when patients are otherwise asymptomatic (Bixler et al. 2016). This is important given that mild‐to‐moderate OSA is highly prevalent in the adult general population, with estimates reported as high as 40% (Young et al. 1997; Bixler et al. 2000; Heeschen et al. 2000; Nieto et al. 2000; Arnardottir et al. 2016). Furthermore, the gold standard treatment for OSA, continuous positive airway pressure (CPAP), has relatively poor compliance (Wolkove et al. 2008), and can be disturbing to both the patient (Wozniak et al. 2014) and their bed partner (McArdle et al. 2001).

While roughly half of all OSA patients also have concomitant hypertension or diabetes (Konecny et al. 2014), there is mixed literature regarding the degree to which OSA is causative, and whether OSA in the mild‐to‐moderate range, specifically, is associated with developing later cardiometabolic dysfunction (Peppard et al. 2000; Reichmuth et al. 2005; O'Connor et al. 2009; Cano‐Pumarega et al. 2011). Importantly, a recent meta‐analysis concluded that CPAP therapy does not significantly alter lipid levels, insulin resistance, inflammatory markers, or the proportion of patients with the metabolic syndrome (Jullian‐Desayes et al. 2015). Thus, given that the severity of AHI alone cannot satisfactorily predict the development of OSA‐associated morbidities, and given limitations of current treatment options, there is a need to identify biomarkers that may help clinicians determine which patients with mild‐to‐moderate OSA will have a worse prognosis, and which intervention may be most beneficial in reducing this risk.

Both OSA and its strongest risk factor, obesity, are independently associated with elevations in systemic inflammation (Vgontzas et al. 2000). Levels of the acute‐phase reactant C‐reactive protein (CRP), synthesized in the liver, rise in response to secretion of interleukins and other cytokines by macrophages and T cells (Chrousos 1995). CRP does not exhibit diurnal rhythmicity like other cytokines, and its levels are dependent upon the severity of overall inflammation in the body. As such, CRP is considered to be a good marker of inflammation and a rough proxy for cardiovascular and metabolic disease risk (Seo 2012); a plasma concentration of ≥3.0 mg/L in adults is considered to put one at high risk (Ridker 2005). We have previously demonstrated that CRP is elevated in a dose–response manner across OSA patients with hypertension, OSA patients without hypertension, and controls, suggesting that CRP may be a clinically useful marker of OSA severity and comorbid cardiovascular problems (Gaines et al. 2015). A more recent study demonstrated that CRP appears to mediate the association between AHI and incident type 2 diabetes (Nagayoshi et al. 2016), suggesting its potential efficacy in discriminating which patients with OSA may have a worse prognosis.

The aim of this preliminary study was to compare the relative utility of AHI versus CRP in detecting, cross‐sectionally, the presence and severity of hypertension and hyperglycemia in middle‐aged adults with mild‐to‐moderate (5 ≤ AHI < 30) obstructive sleep apnea. We hypothesized that CRP would improve our ability to detect cases of mild‐to‐moderate OSA with true cardiometabolic risk that may warrant treatment.

## Methods

### Ethical approval

This research has been reviewed and approved for compliance with the policy of the human subjects Institutional Review Board at Penn State University College of Medicine. Written informed consents were obtained from all participants.

### Participants

The study sample consisted of 60 middle‐aged, predominantly nonobese adults with OSA. Participants were included in analyses if their AHI was greater than or equal to 5 events/h but less than 30 events/h (5 ≤ AHI < 30), and if they provided a fasting blood sample upon awakening. All participants were recruited through advertisements in the local community before being screened in the Sleep Research and Treatment Center at the Penn State Milton S. Hershey Medical Center. Exclusion criteria included a history of diabetes mellitus diagnosis or treatment, rheumatoid arthritis, insomnia, narcolepsy, and use of certain medications (antiglycemics, psychotropics, steroids, sympathomimetics, sympatholytics, or hormone therapy for females). Two participants were regular smokers; excluding or controlling for them did not significantly affect our analyses. All women in the study were postmenopausal (self‐reported absence of menses for at least 12 months or total hysterectomy). All participants were deemed to not have any active illness or infection at the time of their visit, and all participants had CRP levels <7.5 mg/L.

Written informed consents were obtained from all participants. All research protocols were reviewed and approved for compliance with the policy of the human subjects Institutional Review Board at Penn State University College of Medicine.

### Sleep laboratory protocol

During their visit in the laboratory, participants underwent a physical examination, during which height, weight, and waist circumference were recorded. Body mass index (BMI) was calculated (in kg/m^2^).

All participants underwent a single‐night of polysomnography in a sound‐attenuated, light‐ and temperature‐controlled room with a comfortable, bedroom‐like atmosphere. Subjects were continuously monitored for 8 h (22:30–23:00 until 6:30–7:00) using electroencephalogram (EEG), electrooculogram (EOG), and electromyogram (EMG). Respiration was monitored via thermocouple and thoracic/abdominal strain gauges, and hemoglobin oxygen saturation (SpO2) was assessed using a pulse oximeter placed on the index finger. Snoring sounds were monitored via a sensor attached to the throat. All data were recorded using Grass‐Telefactor Gamma Sleep Recording software (Middleton, WI). Visual sleep stage scoring was conducted by a registered polysomnography technologist according to standardized criteria (Rechtschaffen and Kales 1968). Apnea/hypopnea index (number of apneas and hypopneas summed per hour) was ascertained; an apnea was defined as a cessation of airflow with a minimum duration of 10 sec and an associated out‐of‐phase strain gauge movement, while a hypopnea was characterized by a reduction of airflow by approximately 50% with an associated decrease in SpO_2_ of at least 4%.

### Blood draw and assay procedures

Upon awakening (6:30–7:00), fasting blood samples were collected in EDTA‐containing tubes. Plasma was aliquoted into cryotubes after centrifugation and stored at −80°C until assayed. High‐sensitivity CRP was measured via enzyme‐linked immunosorbent assay (ELISA; R&D Systems; Minneapolis, MN). All intra‐ and interassay coefficients of variation were <10%. An additional blood sample (for *n* = 51 participants) was also sent to Quest Diagnostics, Inc. (Madison, NJ) for assessment of fasting glucose.

### Statistical analysis

Hypertension was defined as systolic blood pressure ≥140 mmHg and/or diastolic blood pressure ≥90 mmHg and/or use of antihypertensive medication (National Institutes of Health, 2015), and hyperglycemia as fasting blood glucose ≥100 mg/dL (impaired fasting glucose; (National Institutes of Diabetes and Digestive and Kidney Diseases, 2014). First, we fitted logistic regression models to examine the associations between AHI and CRP with hypertension and hyperglycemia as outcomes, adjusting for age, sex, and BMI. Using these models, we compared the probabilities of having hypertension and hyperglycemia when CRP levels were 0.5 mg/L (normal levels) and 3.0 mg/L (“high risk”) in 50‐year old obese (BMI = 30 kg/m^2^) men with AHI = 15 events/h and women with AHI = 10 events/h; a lower value was used given that women tend to manifest health comorbidities at lower AHI thresholds.

We generated area under the receiver‐operating characteristics (ROC) curves (AUCs). For each outcome, we evaluated the AUCs for three models: Model 1 included demographics (age, sex, BMI) as independent variables; Model 2 added AHI to Model 1; and Model 3 added CRP to Model 2. The estimated risk scores from logistic regression models were used to construct ROC curves.

The statistical confidence level selected for all analyses was *P* < 0.05. Linear regression analyses were performed using the Statistical Package for the Social Sciences (SPSS) version 23.0 (IBM Corp., Armonk, NY), while ROC analyses were performed using Statistical Analysis Software (SAS) 9.4.

## Results

### Sample characteristics

Sociodemographic, sleep, inflammation, and metabolic syndrome characteristics of the sample are presented in Table 1. The sample consisted of 55% men with a mean age of 55.09 ± 5.66 years. The mean BMI of the sample was 29.20 ± 3.67 kg/m^2^, although 41.67% were obese (BMI ≥ 30 kg/m^2^). AHI ranged from 5.13 to 29.15 events/h. CRP levels were, on average, 2.20 ± 1.83 mg/L, with 25% of the sample having CRP levels in the “high‐risk” range (≥ 3.0 mg/L). Average blood pressure was in the prehypertensive range (134.08 ± 17.18 mmHg systolic, 78.48 ± 9.28 mmHg diastolic), and fasting glucose in the prediabetic range (101.29 ± 20.00 mg/dL). When the sample was split by the presence of hypertension and hyperglycemia, CRP levels were significantly elevated in both disorders (*P* < 0.01 and *P* = 0.04, respectively).

**Table 1:** Sociodemographic, sleep, inflammation, and metabolic characteristics of adults with mild‐to‐moderate obstructive sleep apnea

	Full sample		Hypertension			Hyperglycemia		
	Mean (SD) or %	Range	Normotensive (*n* = 31)	Hypertensive (*n* = 29)	*P*	Normoglycemic (*n* = 34)	Hyperglycemic (*n* = 17)	*P*
Age (year)	55.09 (5.66)	44.70–66.40	54.22 (5.35)	56.01 (5.93)	0.22	56.02 (6.16)	53.88 (5.06)	0.22
≥60 year	18.33	−	16.10	20.70	0.75	29.40	0.00	**0.02**
Male (%)	55.00	–	54.80	55.20	0.99	50.00	52.90	0.99
BMI (kg/m^2^)	29.20 (3.67)	22.13–41.75	28.41 (3.28)	30.04 (3.93)	0.08	28.96 (3.95)	30.04 (3.81)	0.35
≥30 kg/m^2^	41.67	–	38.70	44.80	0.79	41.20	52.90	0.55
AHI (events/h)	14.00 (6.69)	5.13–29.15	13.21 (6.48)	14.85 (6.92)	0.35	13.53 (6.88)	15.60 (7.20)	0.32
5 ≤ AHI < 15	56.67		61.30	51.70	0.60	61.80	47.10	0.38
15 ≤ AHI < 30	43.33		38.70	48.30		38.20	52.90	
CRP (mg/L)	2.20 (1.83)	0.12–7.45	1.73 (1.45)	2.69 (2.09)	**0.04**	1.83 (1.56)	3.39 (2.15)	**<0.01**
≥3.0 mg/L	25.00	–	9.70	41.40	**0.01**	17.60	47.10	**0.04**
Systolic BP (mmHg)	134.08 (17.18)	103.00–176.00	120.71 (10.96)	148.38 (9.14)	**<0.01**	133.47 (17.16)	136.76 (18.45)	0.53
Diastolic BP (mmHg)	78.48 (9.28)	58.00–96.00	73.58 (7.54)	83.72 (8.11)	**<0.01**	79.24 (10.39)	77.47 (8.83)	0.55
Fasting glucose (mg/dL)	101.29 (20.00)	59.00–168.00	99.42 (13.10)	102.96 (24.72)	0.53	90.77 (2.28)	122.35 (3.23)	**<0.01**

Data presented as mean (SD) or percentage. Hypertension defined as systolic blood pressure ≥140 mmHg and/or diastolic blood pressure ≥90 mmHg and/or use of antihypertensive medication; hyperglycemia is defined as fasting blood glucose ≥100 mg/dL.

BMI, body mass index; AHI, apnea/hypopnea index; CRP, C‐reactive protein; BP, blood pressure.

### AHI versus CRP in detecting cardiometabolic risk

**Figure 1:**
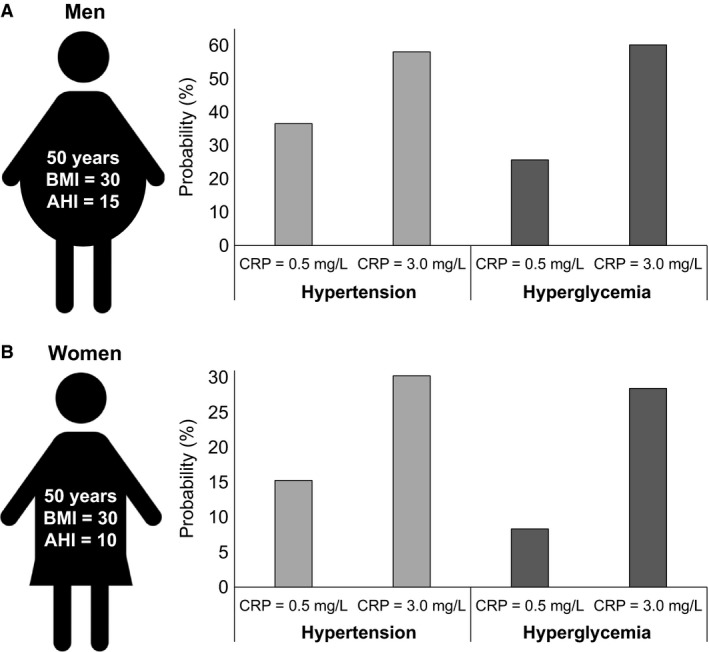
Probabilities of hypertension in “typical” obese, middle‐aged men (A) and women (B) with mild‐to‐moderate OSA when CRP levels are in the healthy (0.5 mg/L) versus “at‐risk” (3.0 mg/L) range.

In logistic regression models adjusting for age, sex, and BMI, CRP was associated with greater odds for hypertension (odds ratio [OR] = 1.42, 95% CI = 0.95–2.12, *P* = 0.086) and hyperglycemia (OR = 1.83, 95% CI = 1.14–2.85, *P* = 0.010) compared to AHI (OR = 1.01, 95% CI = 0.93–1.10, *P* = 0.805; and OR = 1.09, 95% CI = 0.98–1.21, *P* = 0.123, respectively). According to these models, a “typical” obese (BMI = 30 kg/m^2^), middle‐aged (50 years) man with AHI = 15 has a 36.57% probability of having hypertension and 25.69% probability of having hyperglycemia if their CRP = 0.5 mg/L (healthy levels); these probabilities increase to 58.10% and 60.18%, respectively, if their CRP = 3.0 mg/L (“at risk” levels). Similarly, an obese, middle‐aged woman with AHI = 10 has a 15.27% and 8.33% probability of hypertension and hyperglycemia, respectively, if their CRP = 0.5 mg/L; these probabilities increase to 30.24% and 28.43% if their CRP = 3.0 mg/L (Fig. 1).

### Additive discrimination ability of AHI and CRP

**Figure 2:**
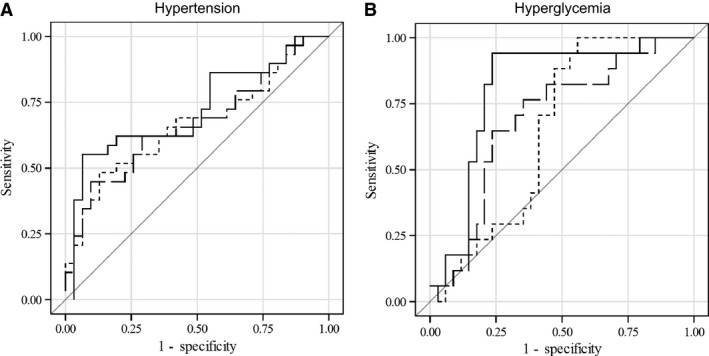
Receiver operating characteristics (ROC) curves for detecting (A) and hyperglycemia (B). Final models (Model 3; age, sex, BMI, AHI, CRP) are represented by solid lines (———). Short dashed lines (‐ ‐ ‐ ‐) represent Model 1 (demographics: age, sex, BMI) as independent variables, while long dashed lines (— — —) represent Model 2 (age, sex, BMI + AHI). Hypertension defined as ≥140 mmHg systolic blood pressure or ≥90 mmHg diastolic blood pressure or use of antihypertensive medication; hyperglycemia defined as ≥100 mg/dL fasting blood glucose.

We then constructed ROC curves (Fig.**** 2) to assess the cumulative ability of our demographic, OSA, and inflammation variables to detect hypertension and hyperglycemia. In terms of identifying hypertension risk, the AUC for the model including demographics only (age, sex, BMI; Model 1) was 0.667 (95% confidence interval [CI] = 0.527–0.808). Incorporating AHI into the model (Model 2) yielded an AUC of 0.670 (95% CI = 0.530–0.810), while adding CRP (Model 3) increased the AUC to 0.721 (95% CI = 0.586–0.856), creating a moderately good model (Table 2; Fig. 2A). In terms of detecting hyperglycemia, the AUC for the model including demographics only was 0.648 (95% CI = 0.500–0.796). Incorporating AHI into the model yielded an AUC of 0.698 (95% CI = 0.547–0.850), and adding CRP increased the AUC to 0.813 (95% CI = 0.668–0.937), which was a significant improvement in accuracy compared to Model 1 (*P* = 0.034; Table 2; Fig. 2B).

**Table 2:** Area under the receiver‐operating characteristics curves comparing AHI and CRP in identifying hypertension and hyperglycemia in adults with mild‐to‐moderate OSA

	AUC (95% CI)
Hypertension (*n* = 60)	
Age, sex, BMI	0.667 (0.527–0.808)
Age, sex, BMI + AHI	0.670 (0.530–0.810)
Age, sex, BMI + AHI + CRP	0.721 (0.586–0.856)
Hyperglycemia (*n* = 51)	
Age, sex, BMI	0.648 (0.500–0.796)
Age, sex, BMI + AHI	0.698 (0.547–0.850)
Age, sex, BMI + AHI + CRP	0.813 (0.668–0.937)

Hypertension defined as ≥140 mmHg systolic blood pressure or ≥ 90 mmHg diastolic blood pressure or use of antihypertensive medication; hyperglycemia defined as ≥fasting blood glucose 100 mg/dL.

AUC, area under the curve; CI, confidence interval; BMI, body mass index; AHI, apnea/hypopnea index; CRP, C‐reactive protein.

## Discussion

In this study, we report that CRP is a stronger biomarker of elevated blood pressure and fasting glucose levels than AHI in middle‐aged adults with mild‐to‐moderate obstructive sleep apnea. CRP levels were also associated with greater odds for having hypertension and hyperglycemia compared to AHI. Furthermore, CRP, in addition to standard clinical measures, yielded moderately good to strong risk models for the disorders. We conclude that including a measure of CRP improves the ability for clinicians to detect cases of mild‐to‐moderate OSA with true cardiometabolic risk, with implications in improving prognosis and treatment options within this clinically gray area.

Given an estimated prevalence of mild‐to‐moderate OSA as high as 40% in the general population (Young et al. 1997; Bixler et al. 2000; Heeschen et al. 2000; Nieto et al. 2000; Arnardottir et al. 2016), the disorder presents a significant public health concern, and identifying its potential impact on cardiometabolic morbidity is of great interest. Two recent reviews (Chowdhuri et al. 2016; McNicholas et al. 2016), however, highlight inconsistent evidence regarding this association with milder forms of OSA. Although half of OSA patients also have hypertension or diabetes (Konecny et al. 2014), the degree to which OSA is causative, particularly within the mild‐to‐moderate range, is unclear (Peppard et al. 2000; Reichmuth et al. 2005; O'Connor et al. 2009; Cano‐Pumarega et al. 2011). Furthermore, there is limited evidence for whether treating mild OSA has a significant impact on cardiovascular events, metabolic disorders, cognitive dysfunction, mood, and traffic accidents. A recent meta‐analysis concluded that while CPAP use may moderately reverse the effects of intermittent hypoxia on sympathetic activity (i.e., reduce blood pressure, heart rate, and catecholamines), CPAP does not significantly alter lipid levels, insulin resistance, inflammatory markers, or the proportion of patients with the metabolic syndrome (Jullian‐Desayes et al. 2015). Moreover, Chirinos and colleagues (Chirinos et al. 2014) demonstrated that combining weight loss with CPAP therapy was significantly more effective than CPAP alone in reducing blood pressure, insulin resistance, triglycerides, and CRP levels over a 24‐week treatment period. Together, these data suggest that not all patients with OSA have, or will develop, cardiometabolic sequelae, and that these sequelae are unlikely to be fully resolved using our current gold standard treatment.

While many studies over the last two decades have reported elevations of certain proteins and hormones in patients with OSA, this is the first study to systematically compare the relative utility of a marker of inflammation against AHI per se in indicating cardiometabolic dysfunction. A joint “biomarker workshop” on sleep health biomarkers sponsored by the National Heart Lung and Blood Institute, National Institute on Aging, and the Sleep Research Society advised that the sleep field is in need of a “unique OSA biomarker signature that is context‐relevant, simple and easy to use” which will help clinicians determine a patient's long‐term prognosis and treatment response (Mullington et al. 2016). A recent editorial by Mehra also highlights the importance of identifying cost‐effective, precise biomarkers for cardiometabolic risk that allow clinicians to potentially identify OSA patients who would be most likely to benefit from therapy (Mehra 2017). CRP is perhaps the most widely studied and consistently elevated biomarker in children, adolescents, and adults with OSA, even independent of obesity and other confounders (Archontogeorgis et al. 2014). Importantly, given that CRP production in the liver is triggered by elevated IL‐6 and other cytokines in response to injury or infection, it is considered to be a good marker of systemic inflammation (Chrousos 1995; Seo 2012). In the case of OSA, obesity,particularly visceral obesity, combined with intermittent hypoxia are thought to be the major mechanisms behind systemic inflammation (Vgontzas et al. 2000). Furthermore, CRP has been shown to be an independent risk factor for future cardiovascular events and metabolic syndrome among both healthy subjects and those with cardiovascular disease (Meier‐Ewert et al. 2001; Heinzer et al. 2015), particularly at levels above 3.0 mg/L (Ridker 2005). Unlike proinflammatory cytokines, CRP levels are not subject to time‐of‐day variation, which may explain why CRP levels are a stronger indicator of health risk (Meier‐Ewert et al. 2001).

Although a relatively routine blood test in certain diagnostic clinics, assessing inflammation in the sleep clinic is rare, if performed at all. While CRP represents a nonspecific marker of inflammation, our study suggests that considering a patient's CRP levels along with other standard sleep clinical measures, age, sex, BMI, and AHI, improves the ability for clinicians to detect cases of mild‐to‐moderate OSA with true cardiometabolic risk. Our findings are corroborated by a recent study suggesting that CRP levels partially mediate the association between AHI and incident type 2 diabetes (Nagayoshi et al. 2016). Another recent paper demonstrated that inflammation was associated with objectively measured daytime sleepiness in patients with OSA, but not with subjectively measured sleepiness, suggesting a link between inflammation and underlying subclinical cardiometabolic dysfunction in OSA (Li et al. 2017). Furthermore, we have previously demonstrated a cross‐sectional, dose–response relationship between CRP levels in patients with OSA and comorbid hypertension compared to nonhypertensive apneics and controls (Gaines et al. 2015). Taken together, these studies suggest that CRP may provide guidance in prognosis and treatment of mild‐to‐moderate OSA given that some individuals with the disorder have, or will later develop, cardiometabolic sequelae. Furthermore, given the limitations of CPAP, a measure of CRP may help clinicians determine how to best treat a patient's AHI while also addressing their increased risk for cardiovascular and metabolic disorders.

There are several limitations to the current study. For one, our sample was relatively nonobese (mean BMI 29.2 kg/m^2^) and restricted to middle‐aged participants (mean 55.1 years, with all participants between the ages of 44 and 66 years). Thus, the sample may not be as generalizable to the general population. However, our age range is representative of peak OSA prevalence in men and women, and the relatively lower BMI range of our sample ensures that our inflammation data is not confounded by morbid obesity. Future studies should examine our hypothesis within a broader age range, particularly given the interesting observation that the association of OSA with cardiovascular problems, such as hypertension, has been shown to decrease with older age (Bixler et al. 2016). Furthermore, given that our study was cross‐sectional, we cannot assign directionality to the associations between mild‐to‐moderate OSA, inflammation, and hypertension/hyperglycemia. Future studies should follow up participants over a period of several years to explore whether CRP levels at baseline may predict future cardiometabolic sequelae, as well as the severity of these sequelae, more so than AHI alone. Understanding the major source of inflammation in OSA (i.e., adiposity vs. intermittent hypoxia) would also provide important insight into the mechanistic link between these associations. Finally, although the sample size was relatively small (*n* = 60), particularly when dichotomized into hypertensive (*n* = 29) and hyperglycemic (*n* = 17) groups, we found that CRP was similarly associated with systolic blood pressure (*β *= 0.29, *P* = 0.06) and fasting glucose levels (*β *= 0.49, *P* = 0.002) significantly better than AHI (both *P* > 0.345) when continuous analyses were conducted.

In summary, our preliminary study suggests that CRP is a stronger marker of hypertension and hyperglycemia compared to AHI in middle‐aged adults with mild‐to‐moderate obstructive sleep apnea. Although mild‐to‐moderate OSA is a rather clinically gray area for many clinicians, we propose that a diagnostic blood test may provide clarity. In this age of personalized medicine, we suggest that considering a patient's CRP concentration, in addition to their demographics and the results of their sleep study, enhances the ability for clinicians to detect cases of OSA with true cardiometabolic risk, improves prognosis, and clarifies which treatment option may be most beneficial to the patient.

## Conflict of Interest

All authors report no biomedical financial interests or potential conflicts of interest.
